# COMPASS: A Computational Pipeline to Identify Linkers Predicting Ubiquitinable PROTAC‐Induced Ternary Complexes

**DOI:** 10.1002/cmdc.70385

**Published:** 2026-07-15

**Authors:** Sébastien Sueron, Sayyed Jalil Mahdizadeh, Eric Chevet, Xavier Guillory, François‐Hugues Porée, Leif A. Eriksson

**Affiliations:** ^1^ ISCR‐UMR CNRS 6226 Faculty of Pharmacy University of Rennes Rennes France; ^2^ INSERM U1242 University of Rennes Rennes France; ^3^ Institute of Complex Molecular Systems (ICMS) Department of Biomedical Engineering, Eindhoven University of Technology Eindhoven Netherlands; ^4^ Department of Chemistry and Molecular Biology University of Gothenburg Göteborg Sweden; ^5^ Centre de Lutte Contre le Cancer Eugène Marquis Rennes France

**Keywords:** degradation, medicinal chemistry, molecular modeling, PROTAC, ternary complex, ubiquitination

## Abstract

PROteolysis TArgeting Chimeras (PROTACs) are bifunctional molecules designed to induce targeted protein degradation by forming a transient ternary complex between an E3 ubiquitin ligase and a protein of interest (POI), leading to the E3‐mediated ubiquitination of the POI and its subsequent proteasomal degradation. Although PROTACs have emerged as highly promising therapeutic tools, rational design remains challenging due to limited structural understanding of the resulting assemblies, the dynamic nature of the ternary interface, and the critical role of the linker. Herein, we present COMPASS (COmputational Modeling of PROTAC Assembly with Structure‐based Screening), a computational pipeline that allows the screening of linker libraries by assessing both ternary complex formation and ubiquitination potential. COMPASS functions as a high‐sensitivity negative filter, identifying linkers that cannot form productive complexes and enabling their elimination before synthesis. Benchmarking against 20 crystallographic structures yielded <6 Å Cα‐RMSD across all systems, outperforming existing methods. Retrospective validation across 8 distinct E3/POI systems (112 PROTACs) yielded 93% recall against degradation endpoints. Discriminative power is strongest when linker geometry is rate‐limiting, a regime complementary to the stability and cooperativity effects that static structural modeling cannot capture.

## Graphical abstract

COMPASS enables structure‐based screening of PROTAC linker libraries by jointly evaluating ternary complex formation and ubiquitination competence. The pipeline accurately recovers experimentally observed ternary complex geometries and identifies linkers unlikely to support productive degradation. By eliminating unproductive candidates before synthesis, COMPASS helps focus experimental efforts on the most promising linker designs.

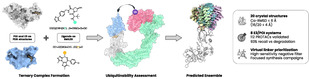

## 1 Introduction

Targeted protein degradation (TPD) has emerged as a promising therapeutic modality in medicinal chemistry, harnessing intrinsic cellular pathways—such as the proteasome, lysosome, and autophagy—to degrade pathogenic proteins. Among TPD modalities, PROteolysis TArgeting Chimeras (PROTACs) stand out as the most clinically advanced approach, exploiting the ubiquitin‐proteasome system (UPS) to achieve degradation of intracellular or transmembrane proteins [1, 2, 3, 4]. The clinical relevance of this strategy has been underscored by the rapid progression of PROTACs into the clinic, with more than 40 compounds currently under clinical evaluation and three currently in phase III trials [5, 6, 7, 8].

PROTACs are bifunctional compounds that orchestrate protein degradation through the UPS, an important eukaryotic machinery dedicated to controlling protein turnover. PROTACs consist of a target protein‐binding warhead and an E3 ubiquitin ligase recruiter, both being connected by a linker. Common linker motifs range from flexible PEG and alkyl chains to conformational scaffolds incorporating aromatic or saturated heterocycles, each conferring distinct geometric and physicochemical properties. This architecture enables the formation of a ternary complex—necessary but not sufficient for degradation. Both stable ternary complex formation and efficient ubiquitin transfer to surface‐exposed lysine residues on the POI are essential for degradation [9, 10, 11, 12, 13]. Ubiquitination efficiency depends on both the geometry of the assembled complex and the accessibility of POI lysine residues to the ubiquitin complex's catalytic machinery. Hence, PROTAC activity is governed not only by binding affinities but also by the structural and spatial constraints imposed by the linker [14].

The linker has emerged as a critical design element, simultaneously modulating protein–protein interactions (PPI) within the ternary complex and influencing physicochemical properties such as permeability and solubility of the resulting PROTACs. Extensive experimental evidence shows that small changes in linker length, rigidity, or exit vector can profoundly alter ternary complex geometry, cooperativity, and degradation outcome [15, 16]. Although certain systems achieve degradation with long, flexible linkers that function through spatial proximity alone rather than direct protein interactions—one recent crystal structure (PDB: 8qjr) reveals ternary complex formation without PPI and with negative cooperativity—most systems require precise linker optimization to achieve productive ternary complex formation and enhanced cooperativity [7, 17, 18].

Despite rapid progress in structural biology and biophysics, rational linker optimization remains a major bottleneck in PROTAC development. Experimental validation of linker chemical space typically requires preparation of large analog libraries exploring exit vectors and linker composition, followed by time‐consuming biophysical and biological evaluation.

While computational modeling offers an attractive route to guide PROTAC design [19, 20, 21], existing approaches for ternary complex prediction are often computationally expensive, rely on multistep energy scoring schemes and software packages, or focus on modeling individual compounds rather than enabling high‐throughput screening. Moreover, nearly all methods assess only the feasibility of ternary complex formation without explicitly evaluating whether a given arrangement is compatible with ubiquitin transfer [22, 23, 24, 25, 26].

To overcome these limitations, we developed COMPASS (COmputational Modeling of PROTAC Assembly with Structure‐based Screening), an automated pipeline designed to screen linker libraries and to identify geometrically feasible, ubiquitination‐competent ternary complexes. COMPASS provides structural predictions to determine whether a given linker can (i) promote formation of a ternary complex and (ii) position the POI for ubiquitination. COMPASS functions as a high‐sensitivity negative filter that identifies linkers unable to form productive ternary complexes, but does not predict degradation efficiency, as downstream factors including cooperativity, ternary complex half‐life, membrane permeability, and cellular context further govern activity. However, by eliminating geometrically infeasible candidates before synthesis, COMPASS enables medicinal chemists to focus resources on linkers with realistic potential for productive ternary complex formation.

## 2 Results

Predicting PROTAC‐induced ternary complex structures is fundamentally a constrained protein–protein docking problem, where the linker defines the accessible conformational space. An effective modeling strategy therefore requires the synergistic application of tools designed for both small‐molecule docking and PPI modeling—methodologies that have individually proven to be reliable in relevant contexts. Existing approaches typically proceed in three steps [27]: (i) generation of PROTAC conformations, (ii) modeling of the protein–protein interaction, and (iii) ternary model construction.

**FIGURE 1:**
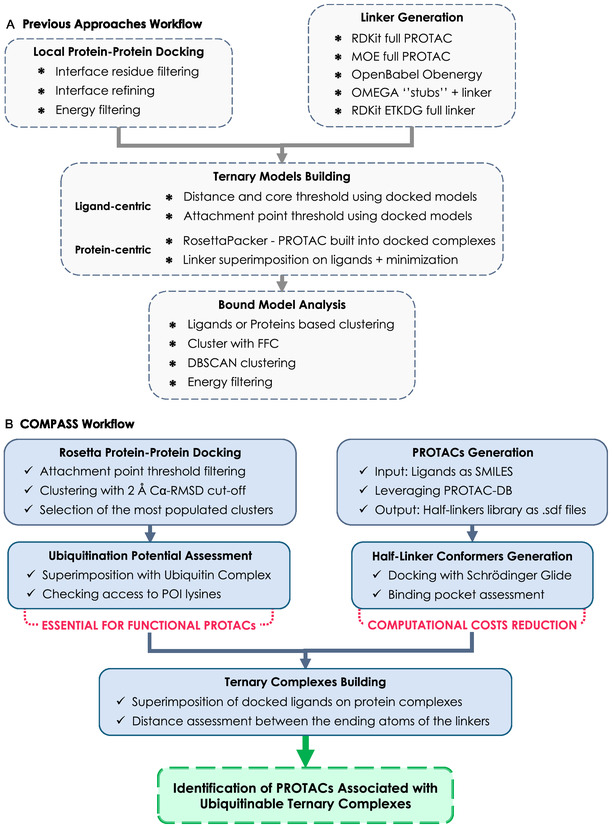
COMPASS Workflow. (A) Comparison of related approaches. Several variants share the same strategy with differences for each step. (B) Main steps of COMPASS to model the dynamics induced by PROTAC ternary complexes. This workflow includes the critical consideration of the ubiquitination potential of a ternary complex.

The COMPASS workflow proceeds in four stages: (i) protein–protein docking to identify potential interface geometries, (ii) validation of ubiquitination potential to ensure functional relevance, (iii) generation of PROTAC conformations using a half‐linker strategy, and (iv) construction of complete ternary models (Figure 1). This step‐by‐step process enables accurate modeling of transient and non‐native protein assemblies while assessing the functional relevance of the predicted complexes, ensuring that the modeled geometries can promote ubiquitin transfer.

### 2.1 Protein–Protein Docking and Clustering of Conformers

The first challenge in modeling PROTAC‐induced ternary complexes is identifying potential protein–protein interfaces between the POI and E3 ligase. Because these assemblies connect two proteins that have not coevolved to interact, extensive sampling is required to capture the accessible conformational landscape. Protein–protein docking is performed with the Monte Carlo‐based RosettaDock software [28, 29], using a local protein–protein docking protocol [30, 31]. Originally developed for antibody–antigen interactions, this protocol presupposes approximate binding patches (akin to epitopes on an antigen and complementarity‐determining regions on an antibody). To overcome the limitations of rigid docking, this protocol allows side‐chain flexibility once a potential interface area is identified. This refinement step optimizes side‐chain orientations without inducing extensive backbone rearrangements, yielding a more realistic interface model [32]. Docking was performed with ligands bound in their respective active sites; COMPASS also accommodates computationally docked ligands or AI‐predicted structures when native crystallographic ligand–protein complexes are unavailable. The E3 ligase is fixed in space while the POI is repositioned to explore potential binding geometries, generating 20,000 candidate conformations per system. Conformations in which a linker could not realistically span the distance between anchor atoms on the respective ligands were discarded (see Methods).

**FIGURE 2:**
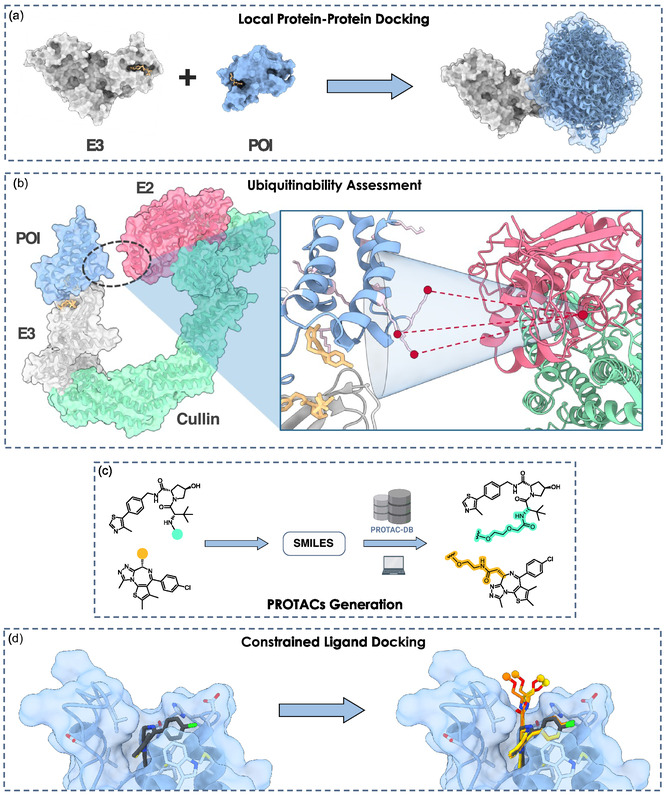
(a) Protein–protein docking using RosettaDock. Generation of protein–protein poses with favorable distance between the two active sites to be able to envisage their binding by a linker. The relevant conformations are then clustered to capture the most favorable contact interfaces between the two proteins. (b) Ubiquitination potential assessment of a ternary complex. After superposition of the entire ubiquitin complex on the E3 ligase, COMPASS verifies that the cysteine residue Cys85 catalyzing the transfer of ubiquitin is correctly positioned and at the right distance from selected lysines of the POI. Inspired by Jofily et al. [38] (c) PROTACs generation. Ligands are provided by the user in SMILES format with a specification of the anchor point. The combination with the PROTAC‐DB allows the generation of a tailored library of PROTAC‐halves. (d) Ligand docking using Schrödinger Maestro. The constraint is fixed on the ligand core with a deviation allocated to 2 Å over the core ligand structure.

Because PROTAC‐induced interfaces are inherently transient and better represented as conformational ensembles, predicted conformations are clustered to identify recurrent interaction interfaces [23, 30, 33, 34]. Conformations are clustered using centroid linkage with a 2 Å RMSD cutoff calculated from residues with any atom within 5 Å of the opposing chain [35, 36]. The optimal number of clusters is determined using Kelley penalty diagrams [37]. Highly populated clusters indicate kinetically accessible interfaces—interface geometries that are frequently revisited during sampling (Figure S1). Clusters are then retained in descending order of population (starting with the most populated) until their cumulative number of total conformations reaches or exceeds 1000, ensuring the inclusion of the most relevant interaction interfaces (Figure 2a). Clusters with equivalent populations at this threshold are retained to avoid arbitrary selection bias.

### 2.2 Validation of the Ubiquitination Potential

A unique feature of COMPASS is its explicit assessment of whether a predicted ternary complex can facilitate ubiquitin transfer, a critical step that conditions UPS‐mediated degradation. This addresses a fundamental limitation of existing computational approaches, which typically focus solely on ternary complex formation without evaluating ubiquitination potential.

To assess ubiquitin transfer competence, we reconstructed the CRL4A (CRBN/DDB1/CUL4A/Rbx1/NEDD8/E2/Ub) and CRL2 (VHL/ElonginC/ElonginB/CUL2/Rbx1/NEDD8/E2/Ub) ubiquitination complexes from available crystallographic data [39, 40, 41, 42]. While these ligases are predominant in the current literature [43, 44], the absence of structural data for other E3 ligases limits the application of this stage to CRBN‐ and VHL‐recruiting PROTACs. For each cluster, the representative structure was superposed onto the corresponding ubiquitination complex (Figure 2b), and three validation criteria were applied: (i) exclusion of any arrangement introducing steric clashes with the ubiquitination machinery; (ii) distance assessments between the catalytic cysteine (Cys85) [45, 46] and POI lysine residues, with lysines beyond ∼60 Å considered inaccessible for ubiquitin transfer [25, 39, 47]; and (iii) a “line of sight” criterion requiring a direct geometric path between the catalytic cysteine and target lysines, excluding residues oriented away from the catalytic site. A margin was allowed to mimic lysine side‐chain flexibility (see Methods). Clusters whose representative structures pass all criteria are considered productive and retained; validation is performed only on representative structures to reduce computational cost, consistent with treating each cluster as a local conformation ensemble. In some cases, no productive cluster may emerge—indicating inherent geometric incompatibility between the POI and E3 system, limited lysine accessibility, or excessive catalytic distance.

Validated clusters can be reused for screening additional linkers or ligand analogs without repeating the protein–protein docking stage. This reusability requires that the new ligand shares sufficient structural similarity with those originally present in the active sites, as the superimposition step relies on conserved core structure and geometry. Ligands with divergent binding modes cannot be reliably screened using precomputed clusters and require de novo docking.

### 2.3 Generating PROTAC Conformations

Effective sampling of PROTAC conformations presents a significant challenge due to the high dimensionality of the conformational landscape. Earlier methods for generating full PROTAC conformations often relied on unconstrained sampling using tools such as RDKit or OpenBabel [27, 48, 49] and produced large conformer libraries—often tens of thousands of structures—many of which were geometrically irrelevant to the protein‐bound context.

COMPASS addresses this dimensionality problem with a fragment‐based approach that samples each half‐linker within its binding environment [34, 50]. Each linker is split at its midpoint (Figure 2c), and the resulting halves are docked individually against the corresponding protein–ligand complex using Schrödinger Glide [51], with a positional constraint (≤2 Å RMSD) on the ligand core (Figure 2d). This constrained docking approach offers several advantages: (i) circumventing the single‐active‐site limitation of standard docking tools, which cannot simultaneously dock a compound spanning two binding pockets, (ii) managing the high dimensionality of PROTACs’ conformational space by sampling each half independently, (iii) enabling each linker segment to adapt to its specific binding environment and explore favorable exit vectors, and (iv) detecting steric clashes early—before full ternary complex reconstruction—thereby reducing computational cost.

To enable high‐throughput screening, we integrated COMPASS with the PROTAC‐DB [52], a database cataloging nearly 3000 unique linkers from the literature. Linkers were split at their midpoint (see Methods), and the resulting half‐linkers were stored in SMILES format with defined anchor points. This representation enables calculation of physicochemical descriptors—such as molecular weight, logP, TPSA, rotatable bonds, and estimated length—allowing users to prefilter candidates before docking. A Python/RDKit script rapidly assembles selected linkers with user‐specified warhead and E3 ligands at designated anchoring atoms (Figure 2c). Because these linkers derive from published PROTACs, their synthetic or commercial accessibility is established, facilitating translation to experimental validation.

### 2.4 Modeling the Ternary Complex

**FIGURE 3:**
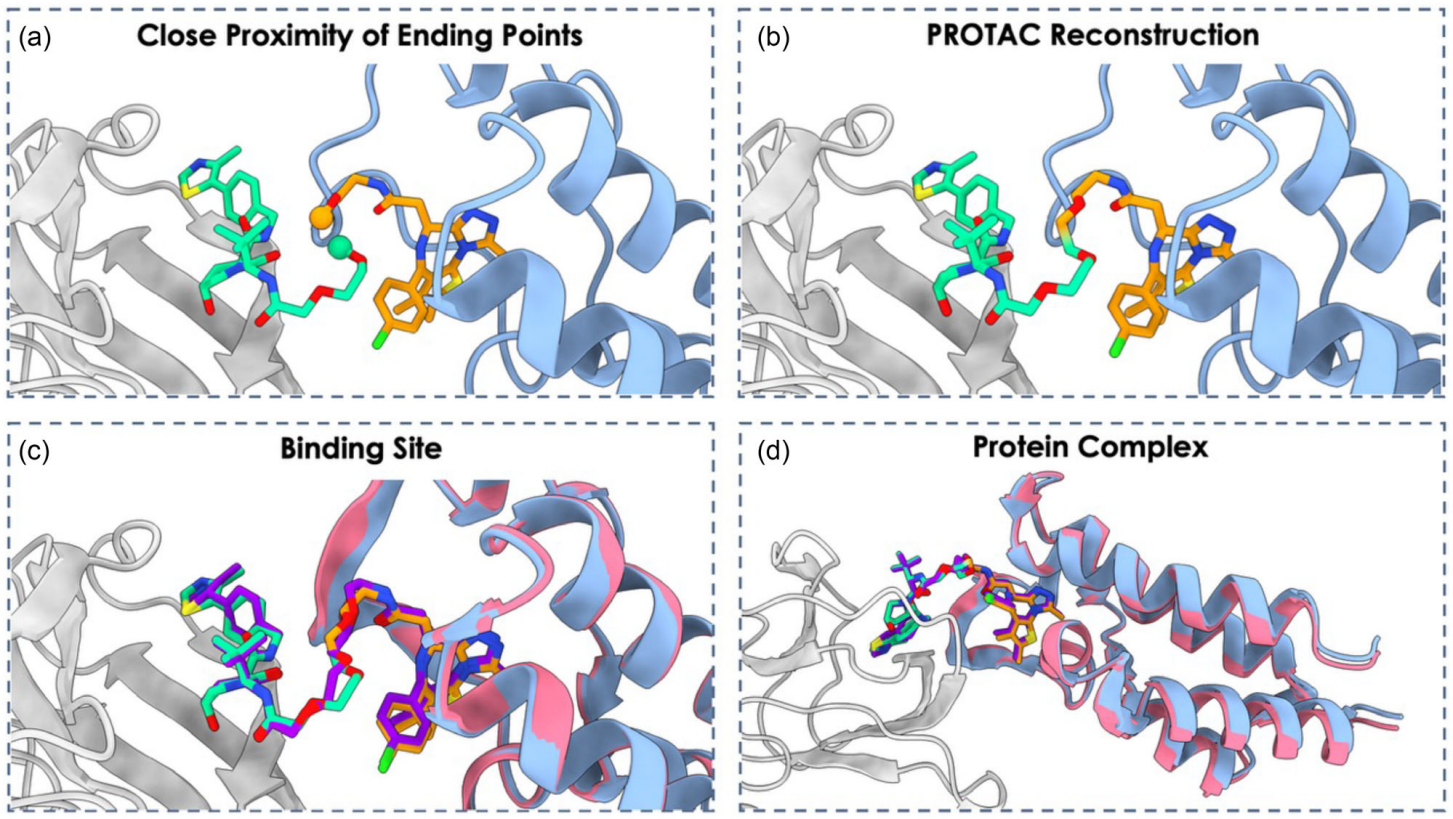
Identification of potential ternary complexes. Superimposition of docked ligands on previously identified productive protein–protein poses: (a) example of a resulting pose with half‐linker end points in close proximity, (b) PROTAC structure after connection and minimization, and (c,d) superimposition of minimized linker with original 5t35 crystal structure.

In the final stage of the modeling workflow, independently generated docked PROTAC half‐linker conformations were combined with pre‐identified protein–protein clusters to construct candidate ternary complexes. Each half‐linker was first superimposed onto its corresponding ligand associated within a cluster, and the resulting assemblies were evaluated based on linker geometry and potential steric clashes with the opposing protein. To enforce chemically plausible connectivity, the distance between the designated terminal atoms of the paired half‐linkers was computed, and only pairs within 0.5–2.5 Å were retained—compatible with an average ∼1.5 Å C—C bond length [53]. Two bond angles were then calculated between each terminal atom and the opposing terminal atom; pairs were rejected if both angles fell below 100° (see Methods). This filtering step enables efficient reconstruction of PROTAC conformations at the protein–protein interface and retains only arrangements likely to form viable ternary complexes (Figure 3). Linkers failing steric, distance or angle criteria were considered geometrically infeasible; those passing were retained as candidate ternary complexes.

### 2.5 Application to a Crystal Structure Benchmark Set

To validate COMPASS, we benchmarked the pipeline against 20 experimentally resolved PROTAC‐induced ternary complex crystal structures retrieved from the RCSB Protein Data Bank repository (PDB IDs: 5t35, 6bn7, 6boy, 6hax, 6hay, 6hr2, 6w7o, 7jto, 7jtp, 7khh, 7pi4, 7q2j, 7s4e, 8bdt, 8dso, 8g1q, 8pc2, 8qvu, 8qw6, 8uh6). This benchmark spans three E3 ligases (VHL, CRBN, cIAP), 10 distinct POIs, and diverse warhead exit vectors and linker chemistries, ensuring comprehensive evaluation across different scenarios (Table 1 and Figure S1).

**TABLE 1:** PROTACs benchmark set and prediction of near‐native structures using Cα‐RMSD. Cα‐RMSD values correspond to the lowest deviation observed among all productive clusters for each system. Time corresponds to the linker docking and ternary assembly stage (step 2.4), excluding protein–protein docking.

PDB	Resolution, Å	E3	POI	Ligand	Cα‐RMSD, Å	Time, min
5t35	2.70	VHL	BRD4^BD2^	MZ1	0.99	11.9
6bn7	3.50	CRBN	BRD4^BD1^	dBET23	0.98	71.5
6boy	3.33	CRBN	BRD4^BD1^	dBET6	0.87	71.2
6hax	2.35	VHL	SMARCA2	PROTAC 2	3.45	10.6
6hay	2.24	VHL	SMARCA2	PROTAC 1	2.44	12.7
6hr2	1.76	VHL	SMARCA4	PROTAC 2	3.45	27.6
6w7o	2.17	cIAP	BTK	BCPyr	1.56	5.1
7jto	1.70	VHL	WDR5	MS33	3.84	18.1
7jtp	2.12	VHL	WDR5	MS67	5.63	2.3
7khh	2.28	VHL	BRD4^BD1^	GNE‐987	1.47	1.8
7pi4	2.24	VHL	FAK	GSK215	0.43	2.9
7q2j	2.50	VHL	WDR5	Homer	4.82	7.8
7s4e	2.25	VHL	SMARCA2	ACBi1	4.53	10.2
8bdt	2.70	VHL	BRD4^BD2^	PROTAC 51	0.37	3.8
8dso	2.33	cIAP	BTK	BCCov	3.13	10.7
8g1q	3.73	VHL	SMARCA4	Compound 1	2.98	9
8pc2	2.80	VHL	FKBP51	SelDeg51	2.05	16
8qvu	2.24	VHL	KRAS^G12D^	ACBI3	4.59	3.6
8qw6	2.20	VHL	KRAS^G12D^	Compound 3	2.79	2.4
8uh6	3.30	CRBN	PTPN2	Cmpd‐1	1.73	9.2

For this benchmarking, COMPASS was applied without the ubiquitination validation step. This choice reflects a fundamental distinction between crystallographic structures and functional ternary complex states [21]. Crystal structures capture thermodynamically stable arrangements under specific experimental conditions—often influenced by lattice contacts and crystallization additives—rather than the conformational ensemble accessible under physiological conditions [54, 55, 56, 57]. Importantly, crystallized conformations need not be productive for ubiquitin transfer, as a geometrically stable ternary complex may still position the POI unfavorably relative to the catalytic machinery. Ignatov et al. noted this discrepancy for several benchmark structures, observing that crystallographic conformations do not consistently satisfy ubiquitination geometry criteria [34]. Because the ubiquitination validation step is designed to identify functionally competent arrangements—a property not captured in static crystallographic snapshots—its inclusion would penalize conformations that accurately match experimental structures yet lack catalytic competence. Accordingly, to fairly assess COMPASS's ability to recover experimentally observed geometries, benchmarking was performed independently on the docking and the linker‐assembly stages.

**FIGURE 4:**
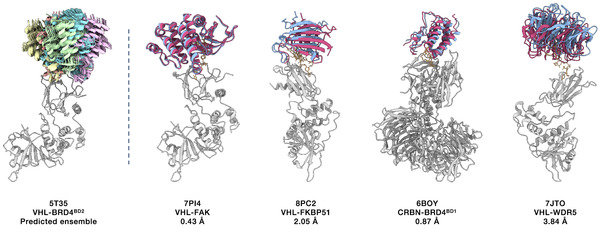
COMPASS predicted ensemble for 5T35 and superimposition of some selected modeled structures with lowest RMSD compared to reference structure. E3 ligases are colored in gray and serve as a reference for superimposing complexes. Modeled ternary complexes are colored in pink while the reference crystal structure is colored in blue. For each structure, the following are provided: PDB‐ID, the partners (E3‐POI) and the Cα‐RMSD (Å).

For each system, COMPASS generated a limited number of productive protein–protein clusters, ranging from 2 to 10 and typically between 2 and 6 per system (Figure 4 and Table S1). These clusters reflect the constrained conformational diversity accessible to a given PROTAC within a specific E3–POI pair. Within each cluster, the configuration exhibiting the greatest number of conformationally valid PROTAC arrangements was selected as the representative structure. This selection strategy acknowledges that PROTAC‐induced ternary complexes occupy shallow, frustrated energy landscapes where multiple conformations coexist at comparable energetic levels. Under such conditions, energy‐based scoring methods struggle to discriminate experimentally observed states from alternative arrangements of similar stability, a limitation consistently noted in prior ternary‐complex modeling studies [23, 30, 41]. Although techniques based on cluster population or calculation of the fraction of protein–protein conformation within a cluster leading to the formation of a ternary complex (i.e., conversion of protein–protein complexes into ternary complexes) can improve predictions, they likewise fail to reliably identify crystallographic conformations using energetic criteria alone [30, 33, 34].

To assess prediction accuracy, representative structures from each cluster were superimposed onto their corresponding crystal structure using the E3 ligase as anchor, and Cα root mean square deviation (Cα‐RMSD) values were calculated using PyMOL (see Methods). Following established criteria by Huang for protein–protein docking and subsequently adopted by Drummond et al. for PROTAC ternary complex modeling, a Cα‐RMSD ≤ 10 Å was considered indicative of successful recovery of a near‐native conformation [22, 58]. For each benchmark system, Table 1 reports the cluster achieving the lowest Cα‐RMSD relative to the experimental structure. Using this criterion, COMPASS successfully recovered near‐native conformations for all 20 systems, with Cα‐RMSD values ranging from 0.37 to 5.63 Å. Ten structures fell below 2.5 Å and 16 below 4 Å, well within accepted thresholds for accurate ternary complex modeling (Figure 4). When compared against ICM [59], MOE [22, 33], and PRosettaC [23] on the 18‐structure subset benchmarked by Rovers et al. [60], COMPASS achieved the lowest RMSD in 12 of 18 cases (Table 2). DockQ scores [61, 62] were computed as an additional interface quality metric (Table S2). Of the 20 benchmark structures, 4 scored as high (>0.80), 8 as medium (0.49–0.80), 7 as acceptable (0.23–0.49), and 1 as incorrect (<0.23), consistent with Cα‐RMSD assessments.

**TABLE 2:** Benchmarking of Cα‐RMSD and comparison of COMPASS with other methodologies.

PDB	COMPASS	ICM	MOE	PRosettaC
5t35	0.99	1.48	8.77	1.85
6bn7	0.98	3.29	5.76	14.01
6boy	0.87	2.86	5.52	18.42
6hax	3.45	3.30	8.52	3.94
6hay	2.44	4.08	7.39	6.37
6hr2	3.45	3.17	4.00	9.82
6w7o	1.56	5.21	3.43	NaN
7jto	3.84	8.25	16.39	24.75
7jtp	5.63	11.59	10.27	8.32
7khh	1.47	3.38	3.22	8.42
7pi4	0.43	16.12	21.58	5.27
7q2j	4.82	4.00	8.14	8.45
7s4e	4.53	3.98	7.38	3.66
8bdt	0.37	1.55	2.19	NaN
8dso	3.13	10.68	11.99	NaN
8pc2	2.05	5.01	19.25	13.93
8qvu	4.59	1.33	9.20	19.51
8qw6	2.79	2.47	5.78	12.08

Protein–protein docking and clustering required 18–24 h per system, depending on protein size. Computational times for the ternary assembly stage ranged from 2 to 72 min per system (Table 1), with most completing in under 15 min on a single compute node (2× Intel Xeon Gold 6130, 32 CPU cores, 96 GB RAM) on the Tetralith computing cluster [63]. These runtimes demonstrate that, once protein–protein clusters are established, COMPASS can efficiently evaluate individual linkers, providing a practical basis for linker‐library screening.

### 2.6 Retrospective Validation Against Published SAR Data

Crystal structure benchmarking demonstrated that COMPASS recovers structurally accurate conformations, but practical utility requires predicting which linkers yield functional outcomes before synthesis and experimental evaluation. To address this question, we retrospectively benchmarked COMPASS against published structure–activity relationship (SAR) data spanning eight distinct E3/POI systems and 112 unique PROTACs, each with reported degradation activity (DC_50_). Each system included both active and inactive compounds, enabling assessment of both recall and false positive rates. A subset of 51 compounds additionally had biophysical measurements of ternary complex formation (e.g., cooperativity, TR‐FRET), allowing separate evaluation against this upstream endpoint.

Unlike the crystal structure benchmark, no experimentally resolved ternary complexes were available for these systems. Input structures were therefore derived from binary protein‐ligand complexes (using either cocrystallized or closely related ligands), apo structures with computationally docked ligands, or Boltz2‐predicted models [64] when no experimental data existed (Table S3). This heterogeneous quality mirrors realistic prospective applications where ternary structural data are unavailable. The ubiquitination validation step—omitted in Section 2.5 to enable fair comparison with crystallographic snapshots—was applied here, as SAR endpoints reflect functional degradation outcomes that depend on productive complex geometry. For each compound, COMPASS produced a binary feasibility classification: a PROTAC was deemed feasible if at least one productive protein–protein cluster satisfying ubiquitination criteria yielded a geometrically viable ternary complex reconstruction. This classification was then compared with experimental outcomes. Across all 112 compounds, COMPASS achieved a recall of 93% against cellular degradation endpoints, with a false positive rate (FPR) of 65% (Table 3). The high recall indicates that active compounds are rarely missed, while the moderate FPR reflects the expected behavior of a geometric filter applied to a biological endpoint governed by additional factors, such as cooperativity or ternary complex half‐life, that are not captured by the static modeling framework used here. Recall remained consistent across all eight systems (73%–100%), with no system showing poor sensitivity, highlighting COMPASS's robustness across diverse E3/POI contexts.

**TABLE 3:** Retrospective SAR validation against degradation across 8 E3/POI systems.^a^.

POI	E3	*n*	*n* Active	Recall, %	FPR, %
TBK1 [65]	VHL	16	11	73	0
SMARCA2 [18]	VHL	11	10	100	100
BRD4 [18]	VHL	6	4	100	100
BTK [66]	CRBN	11	7	100	75
KRAS^G12V^ [67]	VHL	7	2	100	100
LCK [68]	CRBN	16	11	100	100
BRD9 [69]	CRBN	22	18	89	25
JAK2 [70]	CRBN	23	22	96	100
Aggregate		112	85	93	65
SAR Validation against ternary complex formation					
Aggregate		51	47	100	75

a *n* = number of unique PROTACs evaluated per POI/E3 system. *n* Active = number of those compounds reported as active: for the degradation benchmark (upper block), active denotes measurable cellular degradation; for the ternary complex formation benchmark (lower block), active denotes experimentally confirmed ternary complex formation. Recall = fraction of active compounds correctly predicted as active; FPR = fraction of inactive compounds incorrectly predicted as active. Per‐system metrics are computed against cellular degradation endpoints; ternary complex formation metrics are reported only in aggregate. Input structures and structures sources for each system are detailed in Table S2.

System‐level analysis revealed informative patterns. In the VHL‐TBK1 series, COMPASS achieved 0% FPR, correctly classifying all inactive compounds as geometrically infeasible while retaining 73% of active degraders. This case illustrates a regime in which geometric constraints are sufficiently discriminative to separate active from inactive linkers. In the CRBN‐BRD9 system, COMPASS correctly captured activity differences among alkyl linker series differing by single‐carbon increments, demonstrating sensitivity to subtle geometric variations. However, it failed to generate viable reconstructions for two active compounds bearing rigid linker scaffolds within the same ligand pair. These failures are consistent with the constrained conformational space of rigid linkers: viable ternary complex reconstructions require close compatibility between sampled protein–protein geometries and a limited set of linker conformations, making such scaffolds intrinsically more likely to yield no feasible solutions under finite cluster sampling and strict connectivity and angle constraints. Conversely, in systems with high FPR (SMARCA2, KRAS^G12V^, BRD4, JAK2), geometric feasibility does not distinguish active from inactive compounds, consistent with activity being governed by factors beyond ternary complex formation—such as cooperativity, complex half‐life, or cellular context—that lie outside the static modeling framework. Taken together, these results indicate that COMPASS's discriminative power is greatest in systems where linker geometry is rate‐limiting for activity, a regime in which prospective screening is expected to deliver the largest reduction in experimental burden.

We examined the contribution of the ubiquitination validation step to SAR interpretation. For most systems, this step had limited discriminative impact: POIs were relatively small (<300 residues) with broadly distributed surface lysines, and few or no clusters were discarded based on steric clashes or lysine accessibility criteria. However, for the VHL‐TBK1 system (TBK1: 729 residues), ubiquitination modeling proved decisive [65]. One linker identified as inactive in the original study yielded multiple geometrically feasible ternary complex reconstructions when ubiquitination validation was bypassed (Figure S2). Upon explicit modeling of the CRL2^VHL^ ubiquitination machinery, all reconstructions failed due to steric clashes between TBK1 and the E2‐ubiquitin conjugate. This result suggests that inactivity arises not from failure to form a ternary complex per se but from unfavorable spatial arrangement of the POI relative to the catalytic machinery. The original study attributed the inactivity of short linkers (<12 atoms) to steric conflicts preventing ternary complex formation; the ubiquitination modeling presented here supports an alternative explanation in which ternary complexes do form, but position TBK1 unfavorably relative to the E2‐ubiquitin conjugate. This shifts the mechanistic bottleneck from ternary complex assembly to catalytic competence, a distinction that geometric screening alone cannot resolve.

To further probe COMPASS's predictive boundaries, we evaluated the 51‐compound subset with biophysical ternary complex formation data. Against this endpoint, COMPASS achieved 100% recall with a higher FPR of 75% (Table 3). The increase in false positives relative to degradation is expected: ternary complex formation is necessary but not sufficient for degradation—complexes must also achieve adequate stability and half‐life to enable ubiquitination. This limitation motivated an investigation into whether structural metrics derived from COMPASS ensembles could predict experimental cooperativity (*α*), a key determinant of ternary complex stability, for the subset of compounds with available data (*n* = 35). MM‐GBSA scoring [71] was applied to representative ternary complex structures using multiple decomposition schemes, including direct ternary complex energy and derived coupling terms (Figures S3 and S4) and normal mode analysis (NMA) [72] was performed to assess interfacial flexibility (Figure S5). Neither approach yielded significant correlations with experimental cooperativity, consistent with the entropic nature of cooperativity in frustrated, multistate systems that static models cannot capture.

## 3 Discussion

COMPASS addresses a persistent challenge in PROTAC development: predicting productive ternary complexes and prioritizing linkers before synthesis. By integrating protein–protein docking, assessment of ubiquitination potential, and efficient linker sampling, COMPASS provides a systematic framework for navigating the structural landscape of PROTAC‐mediated degradation. Structural benchmarking against 20 crystallographically resolved ternary complexes demonstrated robust performance, with Cα‐RMSD values consistently below 6 Å, with 16 systems below 4 Å, outperforming ICM, MOE, and PRosettaC in 12 of 18 directly comparable cases. Retrospective validation across eight distinct E3/POI systems further establishes COMPASS as a practical tool for linker prioritization: against 112 compounds with cellular degradation data, COMPASS achieved 93% recall; against the 51‐compound subset with biophysical ternary complex formation measurements, recall reached 100%. Together, these benchmarks validate COMPASS as a high‐sensitivity negative filter that identifies linkers incapable of forming productive ternary complexes, enabling their elimination before synthesis.

PROTAC‐induced ternary complexes populate dynamic ensembles with diverse—and distinct—protein–protein interfaces, rather than single stable conformations. This behavior reflects both the structural diversity of protein surfaces and the conformational constraints imposed by the linker [73, 74, 75]. The phenomenon is increasingly understood through protein frustration theory: interfacial residue pairs adopt energetically suboptimal configurations because the POI and E3 ligase have not coevolved to interact [76, 77, 78]. Recent work by Ma et al. demonstrated that interfacial residue pairs in SMARCA2‐VHL complexes adopt energetically suboptimal configurations, where higher cooperativity PROTACs can exhibit a greater proportion of frustrated residue pairs [76]. In such frustrated energy landscapes, multiple conformations coexist at comparable energetic levels, explaining why previous modeling approaches have failed to rank ternary complexes through energetic scoring alone. Experimental evidence supports this dynamic ensemble view. Hydrogen–deuterium exchange mass spectrometry (HDX‐MS) studies reveal conformational plasticity at PROTAC‐mediated interfaces [66, 75, 79]; surface plasmon resonance (SPR) kinetics demonstrate rapid transitions among states [80, 81, 82]; molecular dynamics (MD) simulations capture multiple local substates [41, 75, 83, 84]; and recent cryo‐EM structures capture multiple coexisting conformations within single E3/POI pairs [85]. COMPASS aligns with this understanding by generating and retaining multiple productive clusters per system rather than selecting a unique conformation.

COMPASS provides mechanistic insight beyond binary feasibility classification. The VHL‐TBK1 system illustrates this capacity: one linker yielding multiple geometrically feasible ternary reconstructions was correctly classified as inactive only after explicit modeling of the CRL2 ubiquitination machinery revealed steric clashes with the E2‐ubiquitin conjugate. This result suggests that inactivity arose not from failure to form a ternary complex but from unfavorable POI positioning relative to the catalytic machinery—a distinction that shifts the mechanistic bottleneck from ternary complex assembly to catalytic competence, inaccessible to methods that assess ternary complex formation alone.

A practical advantage of COMPASS lies in its modular workflow. Once protein–protein clusters are established for a given E3/POI pair, new linkers or ligand analogs can be screened without repeating the computationally intensive protein–protein docking stage—provided the new ligands share sufficient structural similarity with those originally present. On a single compute node (2× Intel Xeon Gold 6130, 32 cores, 96 GB RAM), COMPASS screens 600–1000 linkers per week per system (median 9 min, mean 15 min per linker) depending on conformational complexity; scaling to multiple nodes enables complete screening of the PROTAC‐DB (∼3000 linkers) within 1 week, or focused libraries of several hundred linkers within hours. This performance also suggests that even standard desktop computers or laptops with modern processors could potentially run COMPASS for smaller scale analyses, making the tool more accessible to research groups without dedicated high‐performance computing resources. Reliance on established, well‐documented platforms—RosettaDock (freely available to academics) and Schrödinger Glide (widely adopted in medicinal chemistry)—lowers barriers to adoption.

Despite these advances, several limitations do remain in the current implementation. First, the ubiquitination validation stage depends on structural data available for the CRL2 and CRL4A complexes; applying COMPASS to less characterized E3 ligases (e.g., MDM2, DCAF‐type or RNF‐family ligases) requires overcoming this validation step until comparable structural models become available. Second, COMPASS cannot predict cooperativity or ternary complex stability: MM‐GBSA and normal mode analyses yielded no significant correlation with experimental cooperativity values. This is consistent with findings by Ma et al. showing that MM‐GBSA calculations—even when applied to extensive MD ensembles—fail to correlate with measured cooperativity. Their frustration‐based descriptor succeeds because it captures dynamic interfacial states through microsecond‐scale MD sampling, a computational investment that falls outside COMPASS's scope as a rapid screening tool. Future integration with enhanced sampling approaches or machine learning models trained on conformational dynamics may address this gap. Finally, while COMPASS effectively prioritizes linker candidates, biophysical and cell‐based validation remains essential. Indeed, the pipeline predicts geometric feasibility for ternary complex formation and ubiquitination, but not other factors such as protein expression, turnover rates, and/or cellular context that ultimately govern protein degradation efficiency.

## 4 Conclusions

COMPASS provides a systematic framework for modeling PROTAC‐induced ternary complexes and prioritizing linkers before synthesis. By integrating protein–protein docking, an efficient linker conformational exploration strategy, and explicit assessment of ubiquitination potential, the pipeline couples structural validity to catalytic competence—the two structural requirements that any productive linker must satisfy. Benchmarking against 20 crystallographically resolved ternary complexes yielded Cα‐RMSD values consistently below 6 Å, with 16 systems below 4 Å, outperforming existing methods in direct comparisons. Retrospective validation across 8 distinct E3/POI systems (112 PROTACs) achieved 93% recall against cellular degradation endpoints and 100% recall against ternary complex formation data, establishing COMPASS as a high‐sensitivity negative filter: it identifies linkers incapable of forming productive complexes, enabling their elimination before resource‐intensive synthesis and evaluation.

COMPASS does not predict cooperativity, ternary complex stability, or degradation efficiency—factors governed by dynamic and entropic contributions beyond the scope of a rapid screening tool. By systematically eliminating unproductive candidates, the pipeline narrows the experimental search space and accelerates linker optimization. Future integration with enhanced sampling methods, machine learning models trained on conformational dynamics, or ADME prediction tools may extend COMPASS toward stability and cooperativity predictions, further reducing the time and cost of delivering clinically viable PROTAC candidates.

## 5 Methods

### 5.1 Protein–Protein Docking

Protein–protein docking was performed using RosettaDock from the Rosetta software suite (version 2024.09 + release.06b3cf8). E3 ligase and POI structures were extracted from crystallographic coordinates, cleaned with clean_pdb.py, and ligand parameters generated with molfile_to_params.py. Binary complexes were preoriented in PyMOL with ligand binding sites facing one another. For each system, 20,000 candidate conformations were generated with initial perturbations of 5 Å translation and 20° rotation, randomized starting orientation, preserved input side chains, and expanded χ_1_/aromatic‐χ_2_ rotamer sampling. Full command and flag rationales are provided in the Supporting Information (SI‐1). Conformations were filtered by the distance between ligand anchor atoms; for benchmarking, the linker length defined the threshold; for the retrospective SAR validation, the threshold was set by the linker library under evaluation.

### 5.2 Clustering

Filtered conformations were clustered in Schrödinger Maestro Suite (Release 2025‐3) using conformer_cluster.py in centroid linkage mode with a 2 Å RMSD cutoff over interface residues (heavy atoms within 5 Å of the opposing chain—see SI‐2 for specific ASL command). The optimal cluster count was determined automatically via Kelley penalty diagrams. Clusters were retained in descending order of population until cumulative membership reached or exceeded 1000 conformations, ensuring inclusion of the most populated—and thus most frequently sampled—interfaces. Clusters with equivalent populations at this threshold were retained to avoid arbitrary selection bias.

### 5.3 Preparation of PROTAC‐DB

Linkers were extracted from the PROTAC‐DB and cleaved at their midpoint using a custom RDKit script. The central bond along the shortest path between the R1/R2 anchors was cleaved only if: (i) the bond is a C—C single bond, and (ii) the bond is not part of a ring. These conditions prevent heteroatom cleavage which would generate sensitive intermediates (e.g., alcohols, amines) liable to introduce docking artifacts and preserve cyclic linker motifs intact. User‐supplied warhead and E3 ligands, provided in SMILES with dummy‐atom attachment points, were connected to compatible half‐linkers and exported as SDF for docking.

### 5.4 Constrained Ligand Docking

Half‐PROTACs were docked into their respective binary protein‐ligand complexes using Glide (Schrödinger Suite) in Standard Precision (SP) mode with extended sampling. A 2 Å RMSD position constraint was applied to the ligand core. Docking grids were centered on the cocrystallized ligand with increased box dimensions. Poses with Glide docking scores below −6 kcal/mol were retained.

### 5.5 Ubiquitinability Assessment

CRL4A (CRBN/DDB1/CUL4A/Rbx1/NEDD8/E2/Ub) and CRL2 (VHL/ElonginC/ElonginB/CUL2/Rbx1/NEDD8/E2/Ub) were reconstructed from available crystallographic data; multiple DDB1 conformations were incorporated into the CRL4A model to account for BPB‐domain flexibility. For each productive protein–protein cluster, the representative structure was superposed onto the corresponding CRL complex with the E3 ligase as an anchor, and the three criteria described in Section 2.2 were evaluated: (i) steric exclusion, (ii) Cys85(E2)‐to‐POI lysine ε‐nitrogen distance ≤60 Å with a 3 Å flexibility margin, and (iii) line‐of‐sight criterion; lysines oriented away from the catalytic site were excluded. Cys85 of the E2 is used as the sole geometric reference because RING‐type ligases (CRL2/CRL4) catalyze direct E2‐substrate transfer without an E3 catalytic cysteine. The PDB IDs corresponding to the DDB1 conformations used are indicated in Table S4. Clusters satisfying all three were retained.

### 5.6 Ternary Complex Modeling

Docked half‐PROTAC conformations were superimposed onto their bound ligands within validated clusters. Pairs were retained when the distance between terminal atoms fell within 0.5–2.5 Å and at least one of the two terminal–atom bond angles was ≥100° (Section 2.4). Retained pairs were connected and subjected to local minimization to relieve strain at the newly formed bond.

### 5.7 RMSD Calculations

Cα‐RMSD was calculated in PyMOL with the align command, disabling iterative outlier rejection and reporting RMSD without coordinate realignment (Supporting Information‐3).

## Author Contributions

**Sébastien Sueron**: conceptualization, data curation, formal analysis, investigation, methodology, software, visualization, writing – original draft, writing – review and editing. **Sayyed Jalil Mahdizadeh**: supervision, writing – review and editing. **Eric Chevet**: funding acquisition, writing – review and editing. **Xavier Guillory**: funding acquisition, writing – review and editing. **François‐Hugues Porée**: funding acquisition, writing – review and editing. **Leif A. Eriksson**: funding acquisition, resources, supervision, writing – review and editing.

## Funding

This work was supported by Sweden Research Council (Grant 2019‐3684), Ligue Contre le Cancer, Inserm PCSI (Grant 23CP055‐00), Fondation de France (Grant 00130853/WB‐2022‐44441) and Swedish Cancer Foundation (Grant 211447‐Pj).

## Conflicts of Interest

L.A.E. and S.J.M. are co‐founders of ANYO Labs AB (https://www.anyolabs.com). E.C. is founder of Thabor Tx (https://www.thabor‐tx.com/). L.A.E. and E.C. are co‐founders of Exa Noma Therapeutics (https://www.exanoma.com/). The authors declare no conflicts of interest.

## Supporting information

Supplementary Material

## Data Availability

COMPASS, as well as CRL constructs and a complete tutorial, is available at: https://github.com/ssueron/compass.
