# Supplementary material for: COMPASS: A Computational Pipeline to Identify Linkers Predicting Ubiquitinable PROTAC‐Induced Ternary Complexes
Source: ChemMedChem. 2026 Jul 15;21(14):e70385. doi: 10.1002/cmdc.70385 (PMC13372713; doi:10.1002/cmdc.70385)
Supplement: Supplementary file 1 — Supplementary Material [file CMDC-21-e70385-s001.pdf]

COMPASS: A Computational Pipeline to Identify Linkers Predicting Ubiquitinable PROTAC-Induced Ternary Complexes

Sébastien Sueron[1,2,3,*], Sayyed Jalil Mahdizadeh[4], Eric Chevet[2,5], Xavier Guillory[2,5], François-Hugues Porée[1] and Leif A. Eriksson[4,*]

[1]ISCR-UMR CNRS 6226, Faculty of Pharmacy, University of Rennes, F-35000 Rennes, France.
[2]INSERM U1242, University of Rennes, F-35000 Rennes, France.
[3]Present Address: Institute of Complex Molecular Systems (ICMS), Department of Biomedical Engineering, 5600 MB, Eindhoven, The Netherlands.
[4]Department of Chemistry and Molecular Biology, University of Gothenburg, Göteborg 405 30, Sweden.
[5]Centre de Lutte Contre le Cancer Eugène Marquis, F-35000 Rennes, France.

[*]Corresponding Authors: leif.eriksson@chem.gu.se; s.l.sueron@tue.nl

Supplementary Table 1. Within-cluster Cα RMSD metrics

PDB	N retained clusters	Median mean (Å)	Median std (Å)	Median max (Å)
5t35	10	2.82	1.50	6.38
6bn7	10	3.33	1.85	8.29
6boy	5	4.73	2.54	14.78
6hax	10	3.88	1.78	8.06
6hay	11	4.17	1.72	8.59
6hr2	8	4.00	1.87	9.39
6w7o	10	2.99	1.19	5.43
7jto	10	4.28	2.06	9.67
7jtp	9	2.69	1.52	4.07
7khh	11	3.05	1.40	4.40
7pi4	10	3.43	1.43	6.60
7q2j	11	4.58	1.88	9.52
7s4e	10	4.03	2.04	9.59
8bdt	11	2.58	1.65	5.52
8dso	8	3.24	1.73	7.83
8g1q	10	4.01	2.18	9.16
8pc2	10	2.32	1.15	4.71
8qvu	12	2.59	0.94	3.76
8qw6	10	2.59	0.94	3.76
8uh6	10	2.90	1.15	4.62

mean – average Cα RMSD between the cluster's members and the cluster representative
std – standard deviation of those same RMSDs (spread of the cluster around its representative)
max – the worst single member-vs-representative RMSD in that cluster

Supplementary Figure 1. PROTAC structures with colored warheads/linker

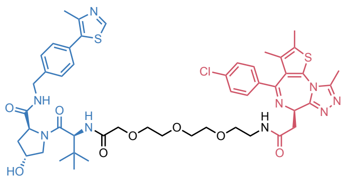

5t35: VHL–MZ1–BRD4^{BD2}

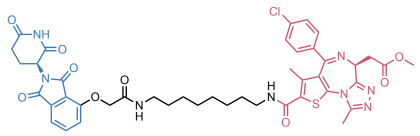

6bn7: CRBN–dBET23–BRD4^{BD1}

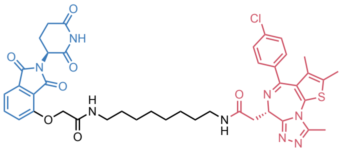

6boy: CRBN–dBET6–BRD4^{BD1}

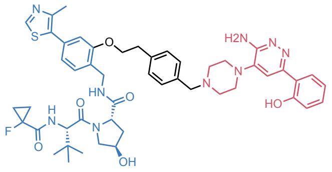

6hax: VHL–PROTAC2–SMARCA2

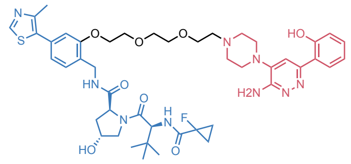

6hax: VHL–PROTAC1–SMARCA2

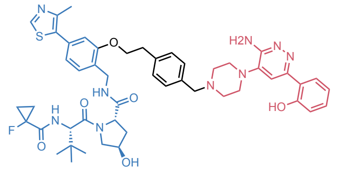

6hr2: VHL–PROTAC2–SMARCA4

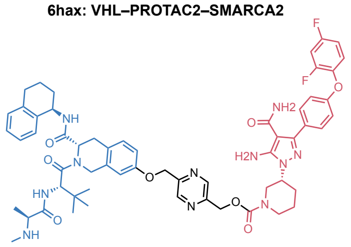

6w7o: cIAP–BCPyr–BTK

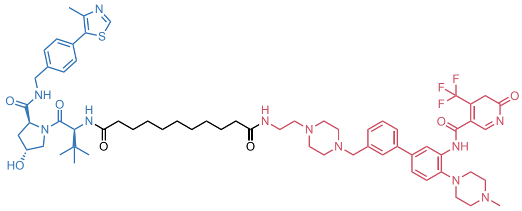

7jto: VHL–MS33–WDR5

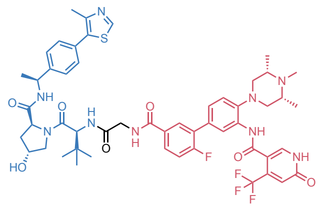

7jtp: VHL–MS67–WDR5

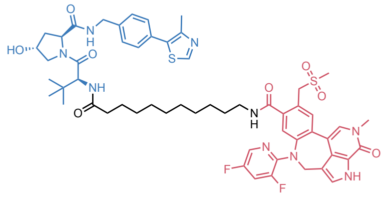

7khh: VHL–GNE987–BRD4^{BD1}

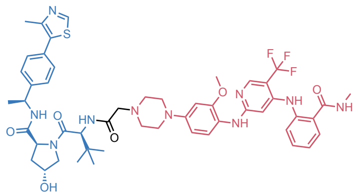

7pi4: VHL–GSK215–FAK

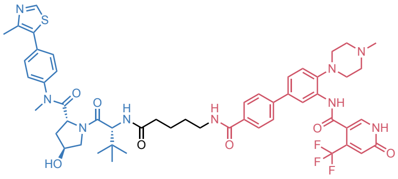

7q2j: VHL–Homer–WDR5

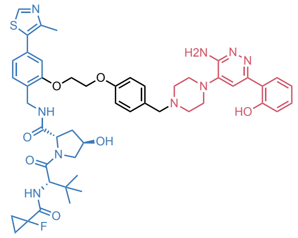

7s4e: VHL–ACBi1–SMARCA2

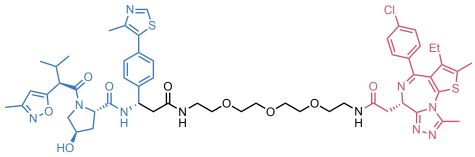

8bdt: VHL–PROTAC51–BRD4^{BD2}

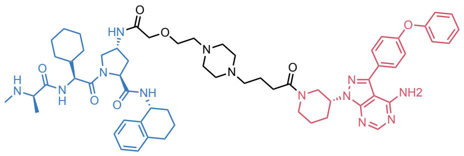

8dso: cIAP–BCCov–BTK

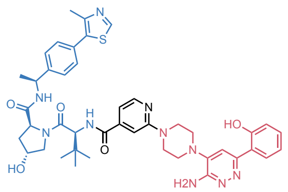

8g1q: VHL–Compound1–SMARCA4

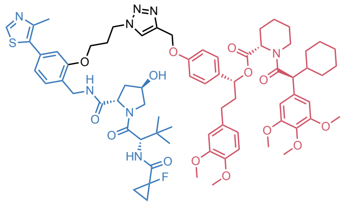

8pc2: VHL–SelDeg51–FKBP51

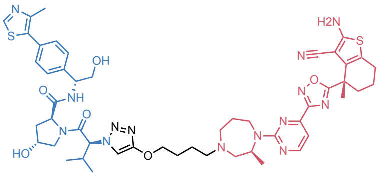

8qvu: VHL–ACBI3–KRAS^{G12D}

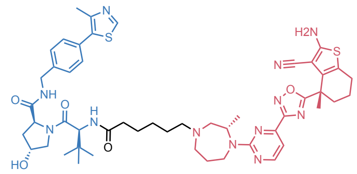

8qw6: VHL–Compound3–KRAS^{G12D}

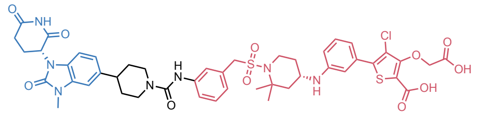

8uh6: CRBN–Cmpd1–PTPN2

Supplementary Table 2. DockQ interface quality scores for COMPASS predictions against crystallographic benchmark structures

PDB	Cα-RMSD (Å)	Dock ↑	Quality	i_RMSD (Å) ↓	f_nat ↑	f_non-nat ↓	F1 ↑
5t35	0.99	0.855	High	0.854	0.909	0.375	0.741
6bn7	0.98	0.756	Medium	1.011	0.737	0.222	0.757
6boy	0.87	0.820	High	0.908	0.857	0.268	0.789
6hax	3.45	0.303	Acceptable	3.336	0.333	0.765	0.276
6hay	2.44	0.472	Acceptable	2.575	0.545	0.647	0.429
6hr2	3.45	0.358	Acceptable	2.627	0.400	0.667	0.364
6w7o	1.56	0.562	Medium	0.562	0.419	0.567	0.426
7jto	3.84	0.448	Acceptable	2.396	0.571	0.000	0.727
7jtp	5.63	0.154	Incorrect	3.622	0.107	0.880	0.113
7khh	1.47	0.738	Medium	1.040	0.739	0.261	0.739
7pi4	0.43	0.953	High	0.351	0.933	0.263	0.824
7q2j	4.82	0.300	Acceptable	3.145	0.353	0.400	0.444
7s4e	4.53	0.297	Acceptable	3.948	0.417	0.762	0.303
8bdt	0.37	0.979	High	0.348	1.000	0.263	0.848
8dso	3.13	0.299	Acceptable	3.608	0.174	0.765	0.200
8g1q	2.98	0.333	Acceptable	3.718	0.200	0.769	0.214
8pc2	2.05	0.528	Medium	2.083	0.545	0.250	0.632
8qvu	4.59	0.230	Acceptable	2.775	0.211	0.692	0.250
8qw6	2.79	0.292	Acceptable	3.437	0.176	0.812	0.182
8uh6	1.73	0.431	Acceptable	2.111	0.317	0.409	0.413

Cα-RMSD: Cα root-mean-square deviation (Å) of the COMPASS model relative to the crystal structure after superposition on the E3 ligase (lowest value among productive clusters; identical to Table 1 in the paper).

DockQ: a composite interface-quality score (0-1) computed from the protein-protein interface; it quantifies how closely the predicted E3-POI interface reproduces the crystallographic interface. Higher values indicate better agreement; the score maps onto the CAPRI quality classes reported in the adjacent column.

Quality: CAPRI classification derived from DockQ scores – Incorrect (<0.23), Acceptable (0.23-0.49), Medium (0.49-0.80), High (>0.80).

i-RMSD: interface RMSD (Å), backbone RMSD over interface residues; lower is better. Interface residues are defined as pairs of residues where any two heavy atoms are within 10 Å of each other.

f_nat: fraction of native residue-residue contacts in the interface recovered in the model; higher is better.

f_non-nat: fraction of model contacts absent from the crystal structure; lower is better.

F1: harmonic mean of contact precision (1 – f_non-nat) and recall (f_nat); higher is better.

A residue-residue contact is defined as any pairs with heavy atoms within 5 Å across the interface.

Supplementary Table 3. Input structures for retrospective SAR validation.

System		Publication (DOI)	Input POI	Input E3
POI	E3		Source [Resolution (Å)]	
TBK1	VHL	10.1021/acs.jmedchem.7b00635	4im0 [2.40]	4w9h [2.10]
SMARCA2	VHL	10.1038/s41467-023-39904-5	6hay[a] [2.24]	
BRD4	VHL	10.1038/s41467-023-39904-5	5t35[a] [2.70]	
BTK	CRBN	10.1073/pnas.1803662115	6mny [2.80]	4ci1 [2.98]
$KRAS^{G12V}$	VHL	10.1021/acs.jmedchem.3c00075	Boltz2[b]	4w9h [2.10]
LCK	CRBN	10.1021/acs.jmedchem.4c00481	Boltz2[b]	4w9h [2.10]
BRD9	CRBN	10.1021/acs.jmedchem.5c01317	5i7x [1.18]	4ci1 [2.98]
JAK2	CRBN	10.1021/acs.jmedchem.5c00831	Boltz2[b]	4ci1 [2.98]

[a] Binary complexes extracted from crystallographic ternary structure, as done for the crystal structure benchmark.
[b] Structure predicted using Boltz2. Uniprot sequences were used as input.

Supplementary Figure 2. TBK1 Ternary Complex Reconstruction and Clash. a) Minimized reconstruction of compound 3d (as referred in original paper) in a VHL-TBK1 complex. b) Exemplified ternary complex dynamics of compound 3d in VHL-TBK1, with several valid ternary complexes. c) Steric clash between a ternary complex of 3d and CRL complex – side view. d) Steric clash – top view.

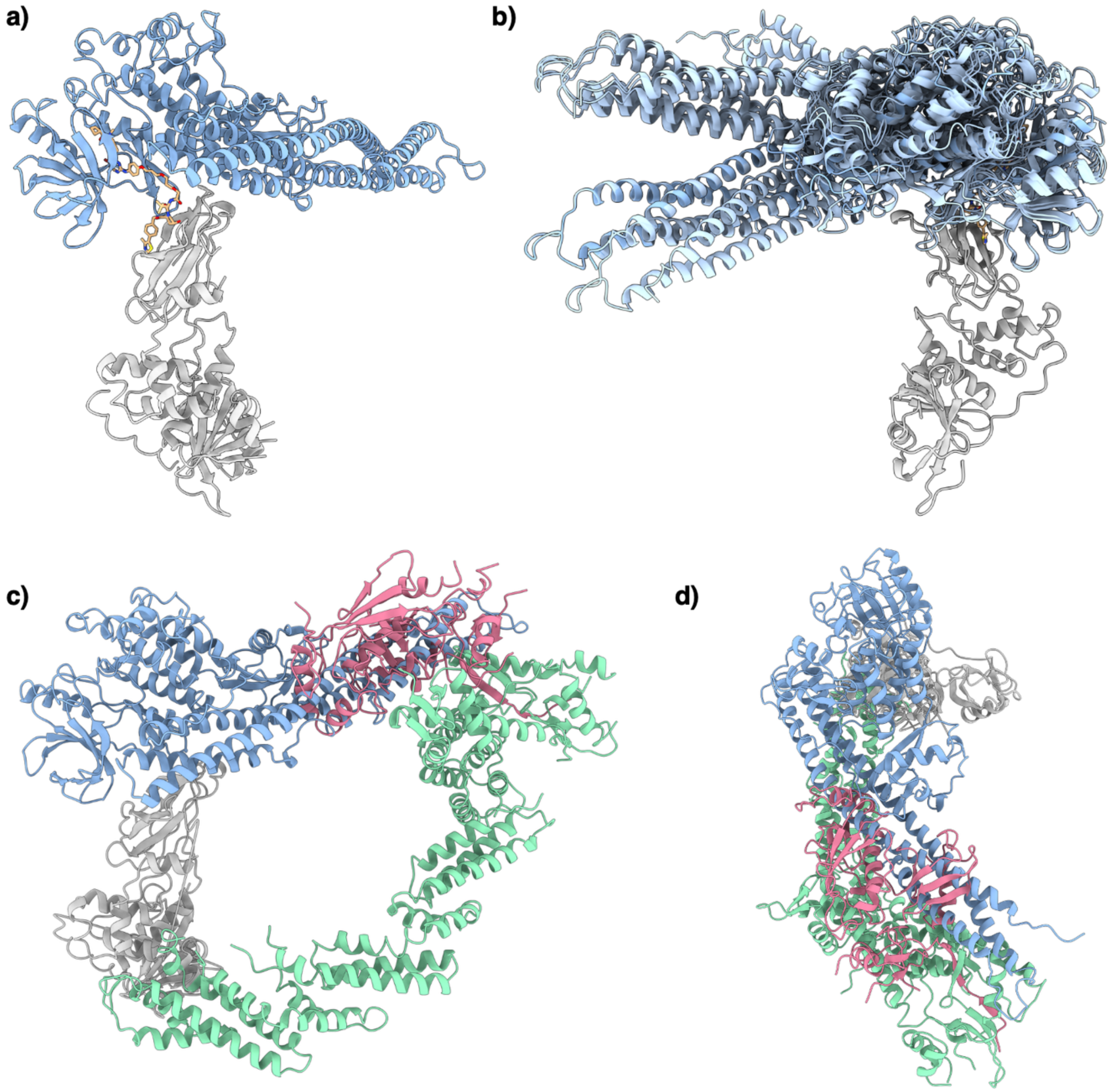

Supplementary Figure 3. MM-GBSA dG_Bind vs Cooperativity by system

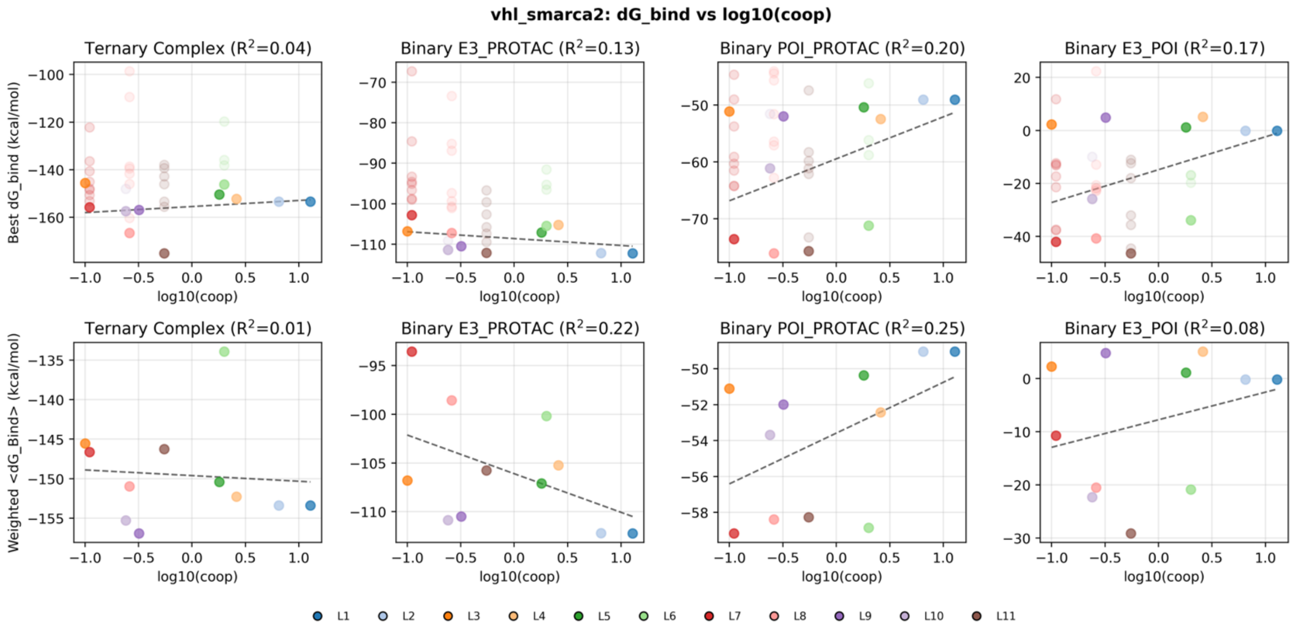

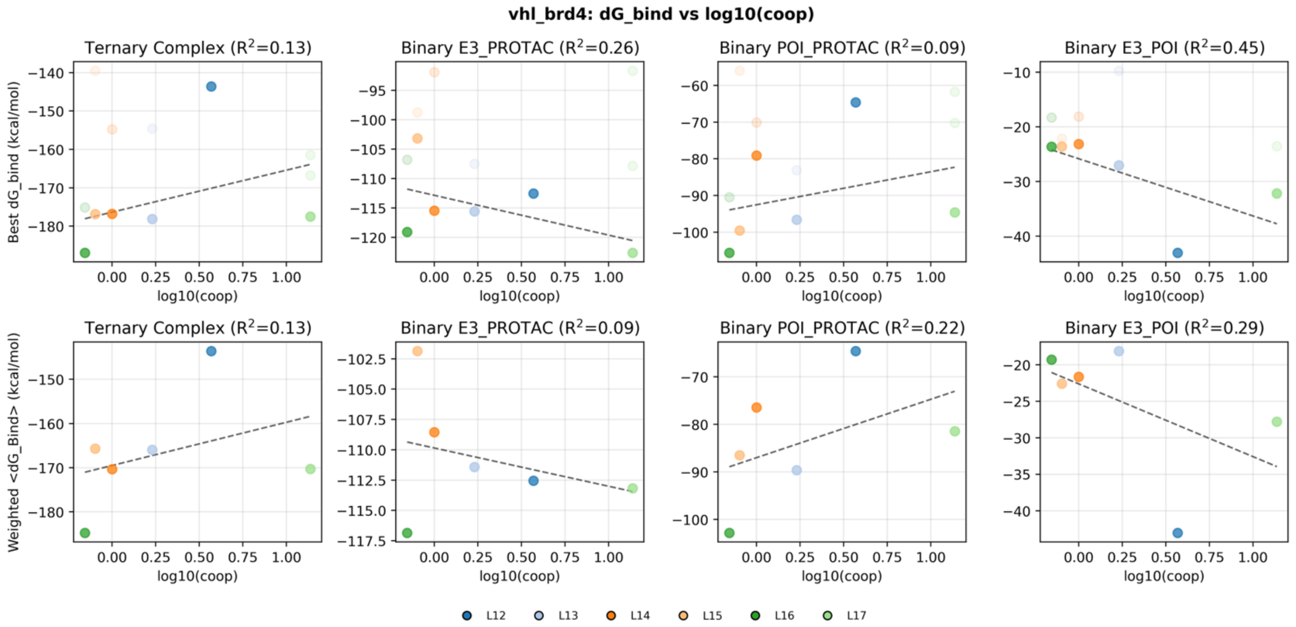

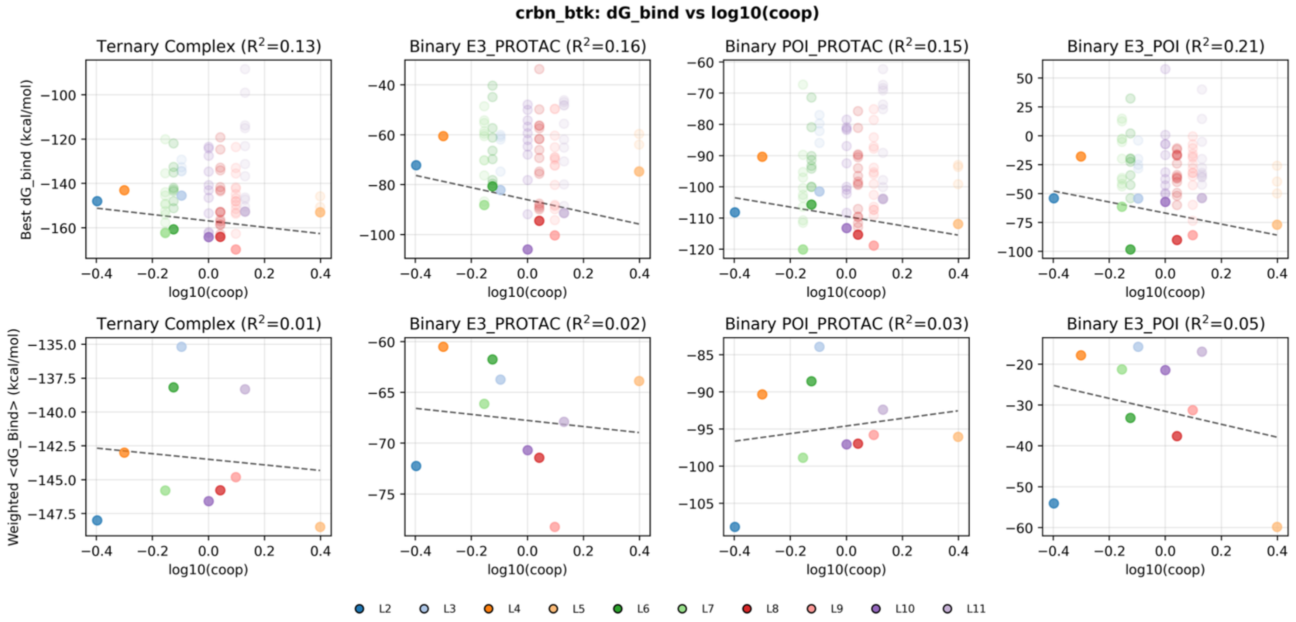

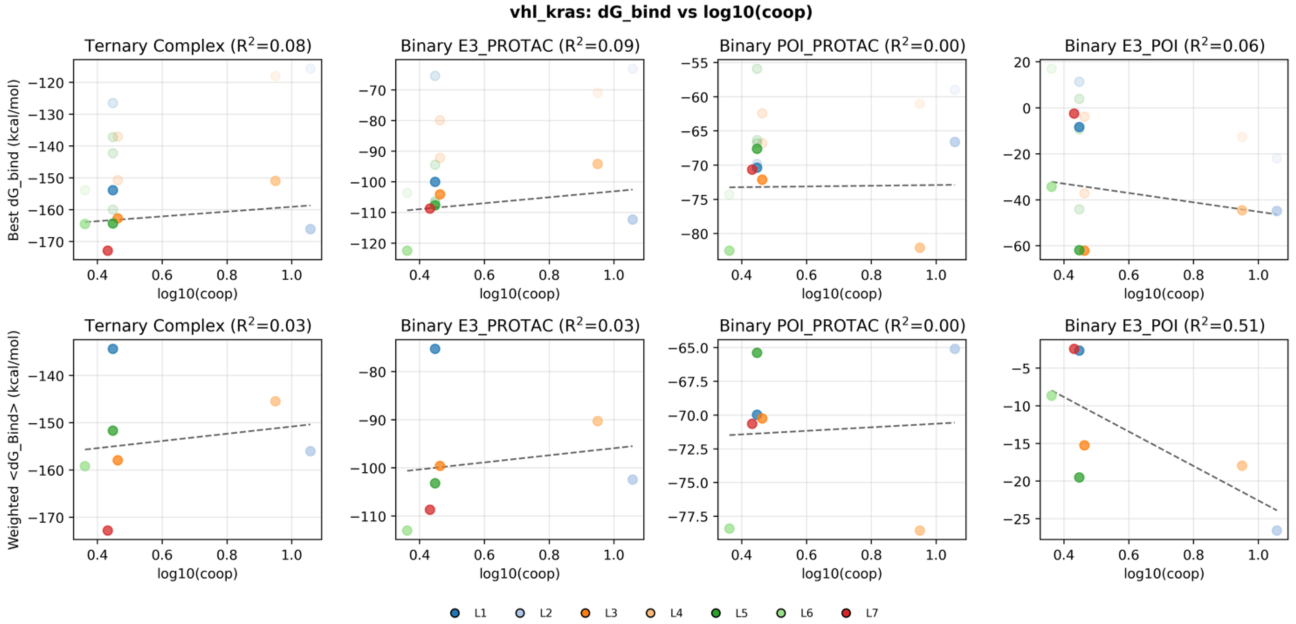

$$\langle \Delta G_{\text{bind}} \rangle = \sum_i w_i \, \Delta G_i, \quad w_i = \frac{N_i}{\sum_j N_j}, \quad N_i: \text{ productive poses in cluster } i$$

Supplementary Figure 4. MM-GBSA Decomposition Terms vs. Cooperativity

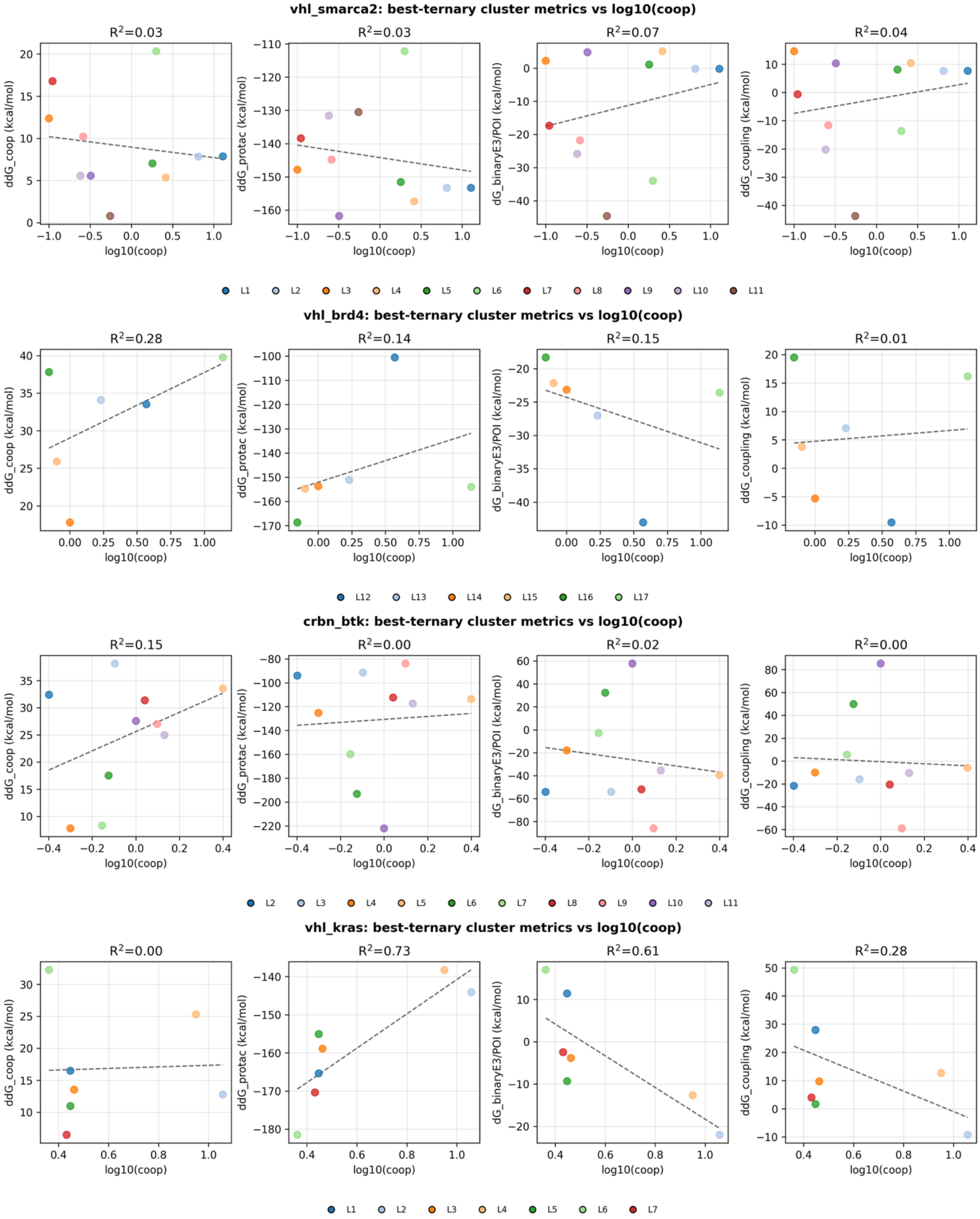

$$\Delta\Delta G_{\text{coop}} = \Delta G_{\text{ternary}} - \Delta G_{\text{binaryE3}} - \Delta G_{\text{binaryPOI}}$$

$$\Delta\Delta G_{\text{coupling}} = \Delta G_{\text{ternary}} - \Delta G_{\text{binaryE3}} - \Delta G_{\text{binaryPOI}} + \Delta G^{\text{apo}}_{\text{binaryE3/POI}}$$

$$\Delta\Delta G_{\text{protac}} = \Delta G_{\text{ternary}} - \Delta G_{\text{binaryE3/POI}}$$

ddG_coop: Excess stabilization of the ternary complex beyond the sum of both binary PROTAC interactions. Captures whether bringing both proteins together via PROTAC is more favorable than expected from independent binding.

ddG_coupling: Three-body coupling term after substracting all pairwise contributions including intrinsic E3-POI affinity.

ddG_protac: PROTAC contribution to complex stability beyond intrinsic protein-protein affinity. Measures how much a degrader impact an interface that may have already some basal affinity.

dG_E3-POI: measures raw PPI energy without PROTAC.

Supplementary Figure 5. NMA - Mean interface RMSF vs Cooperativity by system

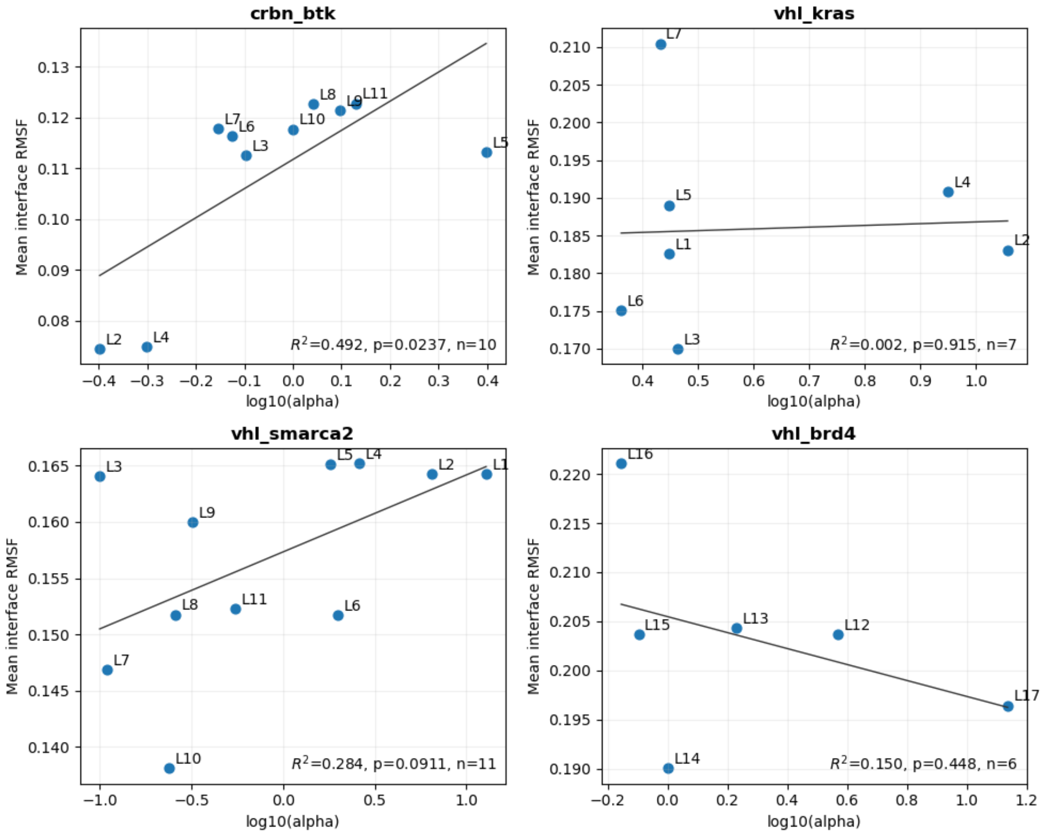

Supplementary Table 4. DDB1 conformations incorporated into the CRL4A model, with source PDB entries

PDB	Resolution (Å)
8ajm	2.83
8d7y	3.40
3e0c	2.41
4e54	2.85
8oiz	2.50
4tz4	3.01
6fcv	2.92
3ei3	2.30
4a0l	7.40
4a08	3.00
2b5l	2.85
6pai	2.90

SI-1 Protein-Protein Docking.

Docking was executed with the following command:

```
./main/source/bin/docking_protocol.linuxgccrelease \
        -s structure_name.pdb \
        -nstruct 20000 \
        -use_input_sc \
        -spin \
        -dock_pert 5 20 \
        -partners CD_AB \
        -ex1 -ex2aro \
        -extra_res_fa ligand1.params ligand2.params \
        -out:file:scorefile score.sc \
        -score:docking_interface_score 1
```

Flag rationales:

-nstruct 20000 generates 20,000 candidate conformations;
-dock_pert 5 20 applies initial perturbations of 5 Å translation and 20° rotation;
-partners CD_AB defines chains C/D as POI and A/B as E3 ligase, CD will move around AB;
-use_input_sc preserves input side-chain conformations;
-spin randomizes the initial orientation around the docking axis;
-ex1 -ex2aro enables expanded rotamer sampling for χ_1 and aromatic χ_2 angles.

SI-2 Clustering Command.

Clustering was performed in the Schrödinger Maestro Suite (Release 2025-3) using the conformer_cluster.py. Filtered protein-protein conformations were clustered on interface residues only, defined as residues with any heavy atom within 5 Å of the opposing chain. Clustering was executed with the following command:

```
conformer_cluster.py protein_complexes.mae \
        -a ”heavy_atoms and fillres ((chain.name A and within 5.0 chain.name C) or (chain.name C and within 5.0 chain.name A))” \
        -in_place \
        -l centroid \
        -n 0 \
        -m 2
```

Flag rationales:

-a defines residues for clustering using a ASL command line: here the heavy atoms within 5 Å of the opposing chain for interface residues
-n 0 enables automatic determination of the optimal cluster count using Kelley penalty diagrams;
-m 2 sets the inter-cluster merge cutoff to 2 Å RMSD;
-l centroid specifies an agglomerative hierarchical centroid linkage - deterministic by nature

The method is deterministic: with fixed inputs, interface selection, and RMSD metric, it yields an identical dendrogram and Kelley partition on every run, independent of seed or order. Centroid linkage can in principle produce a dendrogram inversion, which would make Kelley selection ambiguous; we have empirically confirmed that the MergeDistance curve is monotonic for all 20 benchmarked systems, so no inversion occurs.

SI-3 PyMOL RMSD Command.

align reference and polymer and name CA, modelled and polymer and name CA, cycles=0, transform=0

Flag rationales:

cycles=0 disables iterative outlier rejection, ensuring all Cα atoms contribute to the RMSD calculation transform=0 reports the RMSD without applying alignment transformation, preserving original coordinates
